# Study of PEG-rhG-CSF for the prevention of neutropenia in concurrent chemoradiotherapy for nasopharyngeal carcinoma

**DOI:** 10.1371/journal.pone.0315001

**Published:** 2025-01-15

**Authors:** Lu Yang, Lei Yu, Xue Du, Yu Cui, Guobo Du

**Affiliations:** 1 Affiliated Hospital of North Sichuan Medical College, Nanchong, Sichuan, China; 2 Suining Central Hospital, Suining, Sichuan, China; German Red Cross Blood Service, GERMANY

## Abstract

**Background:**

To study the efficacy and safety of Polyethylene glycolated recombinant human granulocyte colony-stimulating factor (PEG-rhG-CSF) in the prevention of neutropenia during concurrent chemoradiotherapy for nasopharyngeal carcinoma (NPC).

**Methods:**

This is a single-center, prospective, randomized controlled study conducted from June 1, 2021, to October 31, 2022 on patients diagnosed with locally advanced NPC. Participants were divided into an experimental group and a control group. The experimental group received PEG-rhG-CSF injections post-chemotherapy cycles, whereas the control group received standard care without additional intervention. Outcomes assessed included grade 3/4 neutropenia incidence, blood cell count changes, febrile neutropenia rates, delays or interruptions in chemotherapy/radiotherapy due to hematological toxicity, oral mucositis incidents, and bone pain occurrences, comparing these between both groups.

**Results:**

1. 88 patients with locally advanced NPC were included, the incidence of grade 3 neutropenia in the experimental group was lower than that in the control group (P = 0.026); 2. The white blood cell count and neutrophil count in D7, D10, D14, and D21 in the experimental group were higher than those in the control group (P<0.01); 3. The rate of delayed chemotherapy in the experimental group was lower than that in the control group (2.3% vs. 29.5%), P = 0.001; the rate of interruption of radiotherapy in the experimental group was lower than that in the control group (2.3% vs.27.3%), P = 0.003; 4. The incidence of bone pain in the experimental group was 34.1%, of which most were mild bone pain, and no severe bone pain occurred. The leukocyte and neutrophil counts of the patients in the bone pain group were significantly higher than those of the patients in the no bone pain group, P(WBC) = 0.001, P(ANC) = 0.002.

**Conclusions:**

The preventive use of PEG-rhG-CSF decreases the incidence of neutropenia in patients undergoing concurrent chemoradiotherapy for NPC, thereby reducing rates of chemotherapy delays and radiotherapy interruptions, with mild adverse reactions that are tolerable by patients.

## Backgrounds

Nasopharyngeal carcinoma (NPC) is one of the common malignant tumors of the head and neck, originating from the mucosal epithelium of the nasopharynx. In 2020, there were approximately 133,354 new cases of nasopharyngeal carcinoma globally, accounting for 0.7% of all cancers worldwide [1]. Although the overall incidence of nasopharyngeal cancer is low, the geographical distribution of nasopharyngeal cancer is highly imbalanced, with 70% of new cases occurring in East and Southeast Asia [1]. Study Finds Genetic, Racial, and Environmental Factors Are Major Influences on Nasopharyngeal Cancer Pathogenesis [2]. Of these, EBV infection is associated with more than 95% of nasopharyngeal carcinomas in endemic areas [3]. Nasopharyngeal cancer cells are highly sensitive to ionizing radiation, and radiotherapy is the main treatment for early-stage nasopharyngeal cancer [2]. Intensity-modulated radiotherapy (IMRT) is currently the most widely used radiotherapy technique. The 5-year survival rate of stage I patients can be up to 90% with radiation therapy alone [4]. However, most patients with nasopharyngeal carcinoma are already in advanced stages at the time of diagnosis [3], and the therapeutic effect of using only radiation therapy alone is limited. It was found that a platinum-based concurrent radiotherapy regimen can significantly improve the overall prognosis of patients with locally advanced nasopharyngeal cancer [5, 6]. Currently, concurrent chemoradiotherapy has become the standard treatment regimen for locally advanced NPC [7]. However, the simultaneous use of radiotherapy and chemotherapy will also cause the accumulation of toxic reactions, especially the severe neutrophil reduction. Neutropenia is a major cause of treatment interruptions, drug dose reductions, infections, and prolonged hospital stays [8, 9], ultimately adversely affecting the rate of local control of tumors and overall patient survival [10–12].

Granulocyte colony-stimulating factor (G-CSF) is an endogenous hematopoietic growth factor produced by monocytes, fibroblasts and endothelial cells. Its main target cells are neutrophil precursors and mature neutrophils, on the one hand, by inducing the proliferation, differentiation and maturation of neutrophils, increasing the level of neutrophils in the body [13]; On the other hand, G-CSF can further increase the viability and function of mature neutrophils by enhancing the phagocytic and antimicrobial activities of neutrophils, as well as antibody-dependent cell-mediated cytotoxicity, thus contributing to the prevention of febrile neutropenia (FN) [14, 15]. Recombinant human granulocyte-colony stimulating factor (rhG-CSF) is a recombinant form of human G-CSF, which has the same biological activity as endogenous G-CSF [16]. RhG-CSF has been shown in national and international studies to reduce the risk of infection and the morbidity and mortality associated with neutropenia in cancer patients [17], however, due to its predominantly renal clearance and short half-life, rhG-CSF needs to be administered for 7–9 consecutive days to reach therapeutic dose [18]. Repeated injections increase the risk of infection at the injection site, in addition to the fact that continuous daily injections to achieve therapeutic effects require a high degree of patient compliance. For this reason, scientists are further exploring longer-acting agents with longer half-lives and better therapeutic effects. Polyethylene glycolated recombinant human granulocyte colony-stimulating factor (PEG-rhG-CSF), a long-acting rhG-CSF, is produced by selectively cross-linking a 19.6 kDa PEG molecule to the N-terminal end of the rhG-CSF protein. The molecular size of rhG-CSF modified by PEGylation is increased compared to the previous one and, therefore, it is rarely cleared from the body via the kidneys. PEG-rhG-CSF relies almost exclusively on neutrophil receptor-mediated clearance, which is able to reduce plasma clearance with greater intensity [19, 20]. In addition to this, PEG-modified proteins are more resistant to protein hydrolysis compared to unmodified proteins [21], which further prolongs half-life. Not only that, but PEG-rhG-CSF is less immunogenic compared to rhG-CSF, resulting in fewer adverse effects [22–24]. The study confirmed that PEG-rhG-CSF was well tolerated and maintained its efficacy for a long time without serious adverse events [25]. A single dose of PEG-rhG-CSF, on the other hand, ameliorates neutropenia and certain secondary symptoms [26], thus alleviating the pain of repeated rhG-CSF injections and shortening the duration of antitumor therapy in patients. Therefore, PEG-rhG-CSF is more suitable for prophylactic applications.

At present, studies at home and abroad have shown that the prophylactic use of PEG-rhG-CSF can reduce the incidence, duration, and severity of chemotherapy-associated neutropenia in malignant tumors, such as cervical cancer [27], breast cancer [28], lung cancer [29] and lymphoma [30] but there are fewer studies on the efficacy as well as the safety of the prophylactic use of PEG-rhG-CSF during simultaneous radiochemotherapy and they are very rarely associated with head and neck malignant tumors. Therefore, the present study was planned to investigate the efficacy and safety of PEG-rhG-CSF in the prevention of neutropenia during concurrent chemoradiotherapy for nasopharyngeal carcinoma, aiming to ensure that the concurrent chemoradiotherapy is carried out safely, as scheduled, and in sufficient quantity, with a view to providing new clinical references for the treatment of nasopharyngeal carcinoma.

## Materials and methods

### Patient data

**Fig 1:**
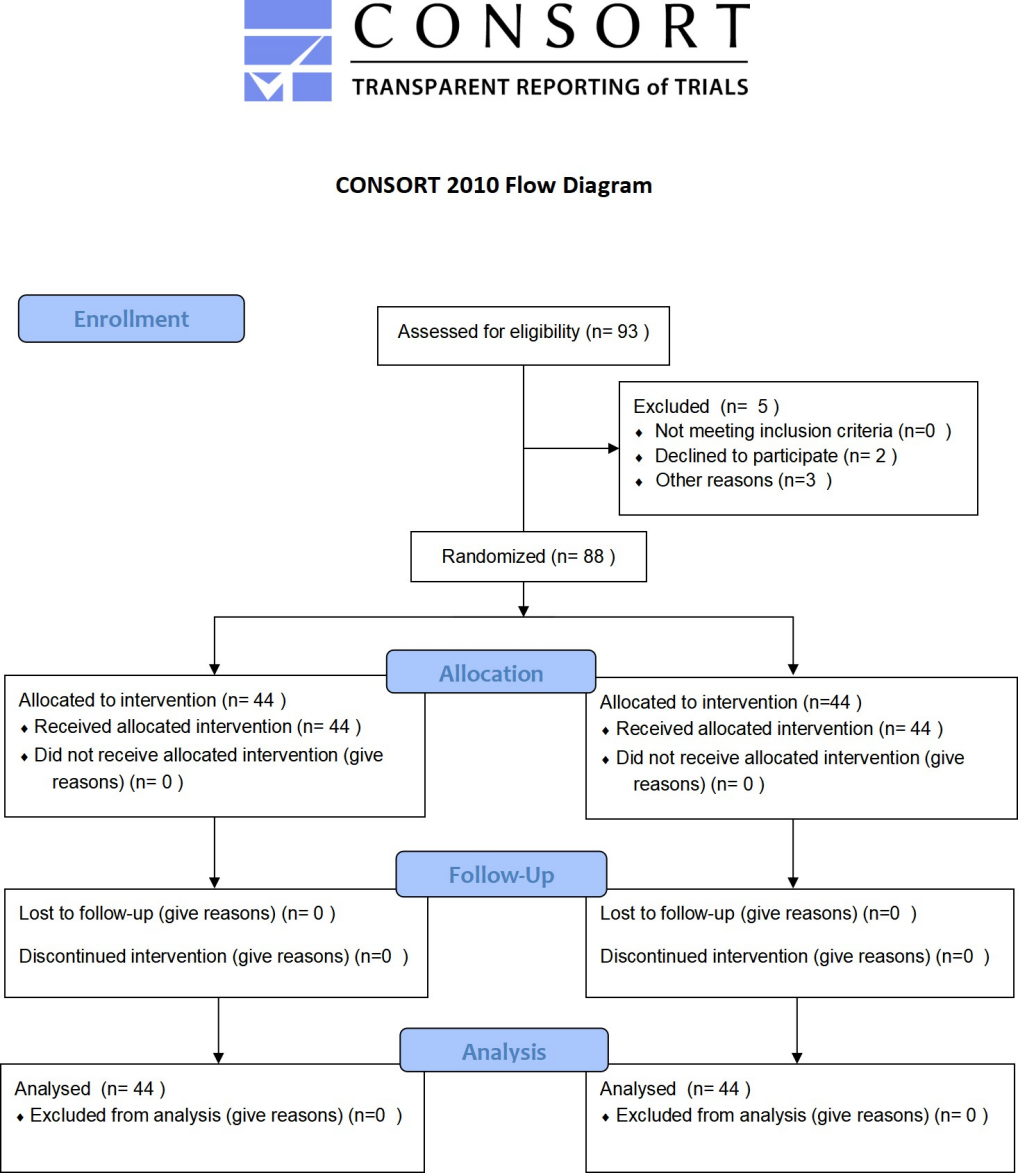
CONSORT participant flow diagram.

The recruitment period for this study extended from June 1, 2021, to October 31, 2022. Patients with locally advanced nasopharyngeal carcinoma (NPC) admitted to our hospital’s Department of Oncology within this timeframe were enrolled, adhering to the AJCC 8th edition staging criteria for nasopharyngeal cancer. Participants were randomly assigned to experimental and control groups at a 1:1 ratio using a computer-based random number table generated by an independent statistician. Simple randomization was employed without blocking or stratification. The allocation sequence was implemented through sequentially numbered, opaque, and sealed envelopes, which were kept by an off-site staff member not involved in the study to ensure allocation concealment until the intervention was assigned. All participating patients provided informed consent in written form, documenting their voluntary participation in the study. It is noteworthy that the study involved only adult patients; hence, no consent from parents or guardians was necessary as minors were not included in the study population. The participant flow through the trial is detailed in the CONSORT flowchart (Fig 1). The research protocol was reviewed and approved by our institutional ethical committee, under the ethical approval number 2021ER054-1 issued by the Medical Ethics Committee of the Affiliated Hospital of North Sichuan Medical College. Additionally, the study has been registered with the Chinese Clinical Trial Registry, bearing registration number ChiCTR2100048522, with the registration date being September 7, 2021.

### Inclusion criteria

1. Patients with nasopharyngeal cancer confirmed by pathology;
2. Clinical stage is locally advanced.
3. Age: age 18–70 years old;
4. Pre-chemotherapy leukocyte (WBC) count ≥3.0 x 10^9^/L, neutrophil count (ANC)≥2.0 x 10^9^/L, platelet (PLT) ≥100 x 10^9^/L, hemoglobin (HB)≥100g / L;
5. The KPS score was> 75;
6. No hematological disease;
7. No other malignant tumors;
8. No previous radiation therapy treatment;
9. Consent to the enrollment and voluntarily signed the informed consent form;

The NPC staging criteria were used according to the AJCC eighth edition of the NPC clinical staging criteria.

### Exclusion criteria

1. Drug allergy;
2. Patients are generally in poor condition and cannot tolerate treatment;

### Procedure

All patients were treated with Intensity-modulated radiotherapy (IMRT) using the Elekta Synery medical linear gas pedal 6MV X-ray technique; 95% of the planned tumor volume (P-GTV) was prescribed to receive a dose of 67–72 Gy, 5 times/week. All patients were treated with simultaneous single-agent cisplatin chemotherapy at a cisplatin dose of 80 mg/m^2^ by intravenous infusion over d1-d4 [31]. One cycle of chemotherapy was given in 21 days, and two cycles of cisplatin chemotherapy were synchronized during radiotherapy. The experimental group received a single subcutaneous injection of 6 mg of PEG-rhG-CSF 24-48h after each cycle of chemotherapy [32]. The control group did not receive any special treatment after chemotherapy.

If patients in both groups had a leukocyte count <2.0×10^9^ /L or a neutrophil count <1.0×10^9^ /L during radiotherapy, radiotherapy was suspended, and rhG-CSF was given subcutaneously until the leukocyte count was ≥4.0×10^9^/L or the absolute neutrophil count was ≥2.0×10^9^/L, and radiotherapy was resumed. If the leukocyte count was <3.0×10^9^/L or the neutrophil count was <2.0×10^9^/L before the second cycle of chemotherapy in both groups, chemotherapy was delayed, and rhG-CSF was administered subcutaneously until the leukocyte count was ≥3.0×10^9^/L or the absolute neutrophil count was ≥2.0×10^9^/L, and chemotherapy was resumed.

### Endpoints

Primary endpoint: The incidence of grade 3 / 4 neutropenia during concurrent chemoradiotherapy.

Secondary endpoint: (1) changes in leukocyte, neutrophil, hemoglobin, and platelet counts on days 7, 10, 14, and 21 of chemotherapy; (2) incidence of FN in the patients during concurrent chemoradiotherapy; (3) rates of delayed chemotherapy and interruption of radiotherapy due to hematologic toxic reactions during concurrent chemoradiotherapy; (4) incidence of oral mucositis during concurrent chemoradiotherapy; (5) incidence of bone pain in the experimental group during concurrent chemoradiotherapy. Among them, oral mucositis was graded according to the WHO mucosal reaction grading criteria.

### Sample size estimation

The formula for sample size estimation used in this study was the sample size estimation method for measurement data in clinical trials. The statistical criteria were set with a significance level α = 0.05 and a power of the test (1-β) = 0.950, where Zα = 1.960 and Zβ = 1.645. The margin of error and the overall variance were calculated from preliminary experimental data, with a margin of error of 2.38 and an overall variance of 4.69. Using the formula *N = 2(Zα+ Zβ)*^*2*^*δ*^*2*^*/d*^*2*^, where δ represents the overall standard deviation and d represents the margin of error, the sample size per group (N1) was approximately calculated to be 22. Considering potential dropouts, it was necessary to increase the sample size by 10%-20%. For this experiment, a 20% attrition rate was taken into account, resulting in a minimum of 27 cases per group. To ensure trial rigor and address potential attrition, statistical power, and robustness concerns, we decided to enroll 44 cases per group, totaling 88 subjects.

### Statistical analysis

IBM SPSS 26.0 software was used to statistically analyze the data. Continuous measures with normal distribution were expressed as mean ± standard deviation, independent samples t-test was used to compare the differences between groups, and paired t-test was used to compare the differences within groups. Repeated measures ANOVA was employed to analyze data from multiple measurements taken on the same subjects. Categorical data were expressed as component ratios, and the chi-square test and Fisher’s exact test were used to compare the differences between groups of categorical data. Differences were considered statistically significant at P < 0.05.

## Results

### Patient characteristics

A total of 88 cases of eligible nasopharyngeal cancer patients were included, of which 60 cases were male patients and 28 cases were female patients; the age was between (21–70) years old and the median age was 53 years old. Forty-four cases in the experimental group and 44 cases in the control group were included, the median age of the experimental group was 52.5 years old and the median age of the control group was 53 years old. The specific baseline clinical data are shown in Table 1.

**Table 1:** General clinical data of 88 patients with NPC.

characteristics	experimental group (n = 44)	control group (n = 44)
Age (year)	51.16 ± 9.93	51.93 ± 11.04
Gender		
male	28(63.6%)	32(72.7%)
female	16(36.4%)	12(27.3%)
Stage		
T		
1	7(15.9%)	2(4.5%)
2	15(34.1%)	14(31.8%)
3	14(31.8%)	17(38.6%)
4	8(18.2%)	11(25%)
N		
0	4(9.1%)	1(2.3%)
1	2(4.5%)	6(13.6%)
2	27(61.4%)	28(63.6%)
3	11(25.0%)	9(20.5%)
Stage		
III	25(56.8%)	24(54.5%)
IV	19(43.2%)	20(45.5%)
WBC before concurrent chemoradiotherapy (×10^9^/L)	6.10±1.87	5.82±1.67
ANC before concurrent chemoradiotherapy (×10^9^/L)	4.25±1.59	4.00±1.54
PLT before concurrent chemoradiotherapy (×10^9^/L)	193.09±55.81	192.73±67.45
HB before concurrent chemoradiotherapy (g/L)	138.07±47.86	133.93±26.93

### Primary endpoint

Among the 44 patients in the experimental group who used PEG-rhG-CSF prophylactically, no patient developed grade 3/4 neutropenia; among the 44 patients in the control group, 6 patients developed grade 3 neutropenia, and no patient developed grade 4 neutropenia, and the incidence rate of grade 3 neutropenia was 13.6% (6/44), P = 0.026, with a statistically significant difference in the incidence rate of grade 3 neutropenia between the experimental group and the control group (Table 2).

**Table 2:** Incidence of grade 3 neutropenia during concurrent chemoradiotherapy.

Indicators	experimental group (n = 44)	control group (n = 44)	P
Grade 3 neutropenia (%)	0	13.6	0.026

### Secondary endpoint

#### Changes in the blood cells

**Fig 2:**
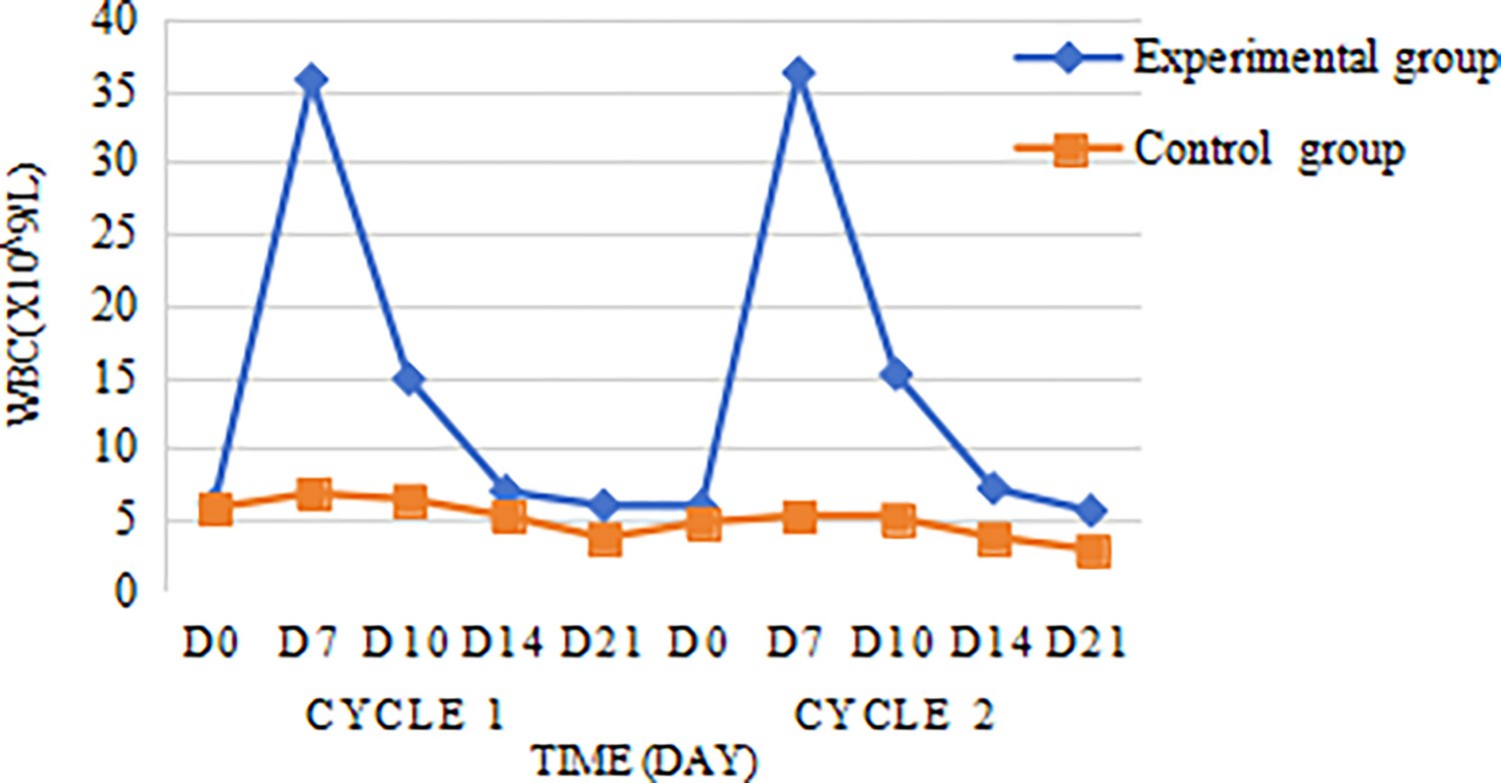
Changes in the white blood cell count.

**Fig 3:**
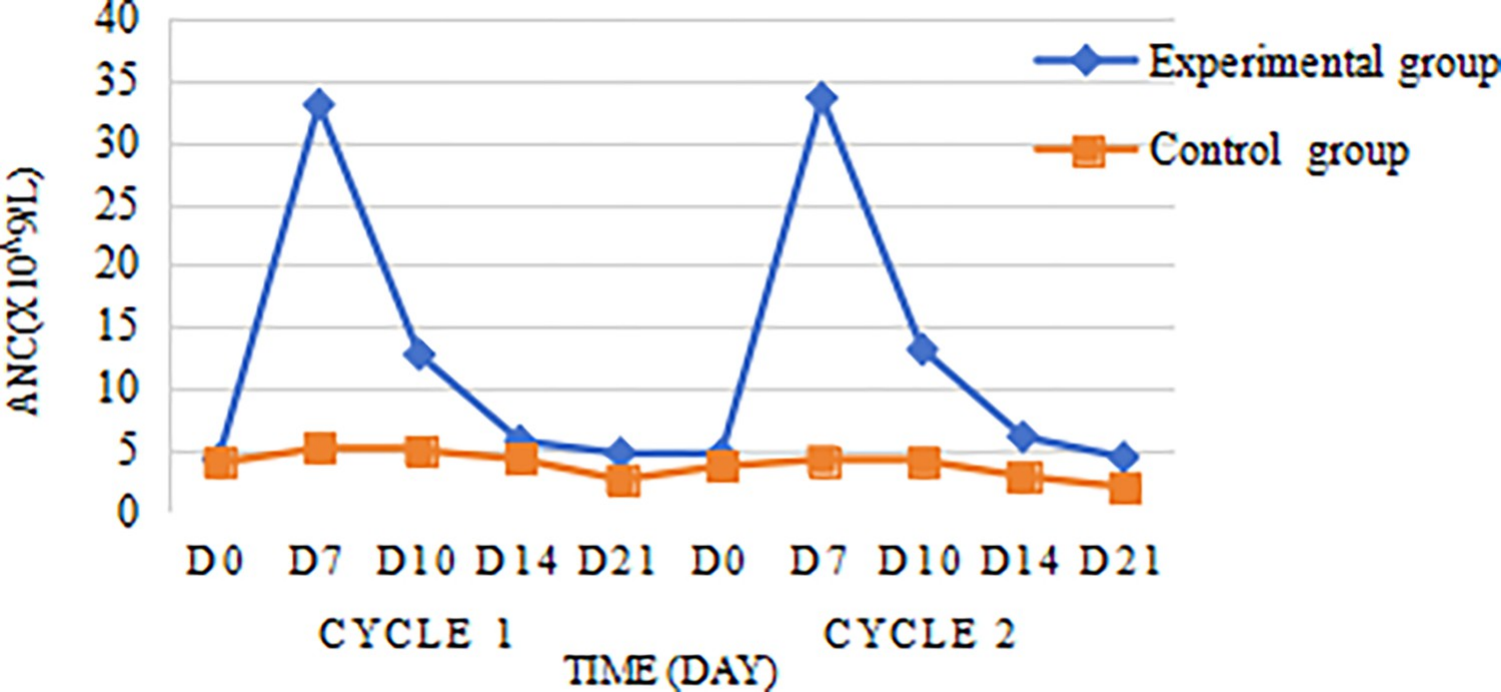
Changes in the neutrophil count.

Leukocyte count and neutrophil count of the experimental group reached the highest value on the 7th day after chemotherapy, and the peak of leukocyte in the control group also appeared on the 7th day after chemotherapy, the leukocyte count of the experimental group decreased significantly after the 7th day, and then decreased slowly after the 14th day, and then decreased to the lowest value on the 21st day, which was (5.97±1.92)×10^9^/L; in the control group, the WBC in the experimental group decreased slowly after day 7, and then decreased to the lowest on day 21, which was (3.71±1.11)×10^9^/L. The WBC counts and ANC counts of the experimental group were higher than those of the control group at D7, D10, D14, and D21 (Figs 2 and 3). Further analysis using repeated measures analysis of variance (RM-ANOVA) revealed significant differences in ANC between the experimental and control groups (F = 205.04, P < 0.0001). Similarly, there were also highly significant differences among the various time points (F = 109.57, P < 0.0001). Additionally, a significant interaction effect was observed between group and time (F = 87.50, P < 0.0001), indicating that the changes in ANC over time differed significantly between the experimental and control groups (Table 3).

**Table 3:** Repeated measures analysis of variance (RM-ANOVA) of ANC changes between experimental and control groups during concurrent chemoradiotherapy.

	ANC (Mean±SD, ×10^9^/L)					Time Effect		Between-Group Effect		Interaction Between Time and Group	
Indicators	D0	D7	D10	D14	D21	F	P	F	P	F	P
Experimental group	4.25±1.59	33.00±16.85	12.75±5.06	5.76±1.90	4.76±1.73	109.57	<0.0001	205.04	<0.0001	87.50	<0.0001
Control group	4.00±1.54	5.17±1.76	5.00±1.88	4.28±2.10	2.78±1.00						

**Fig 4:**
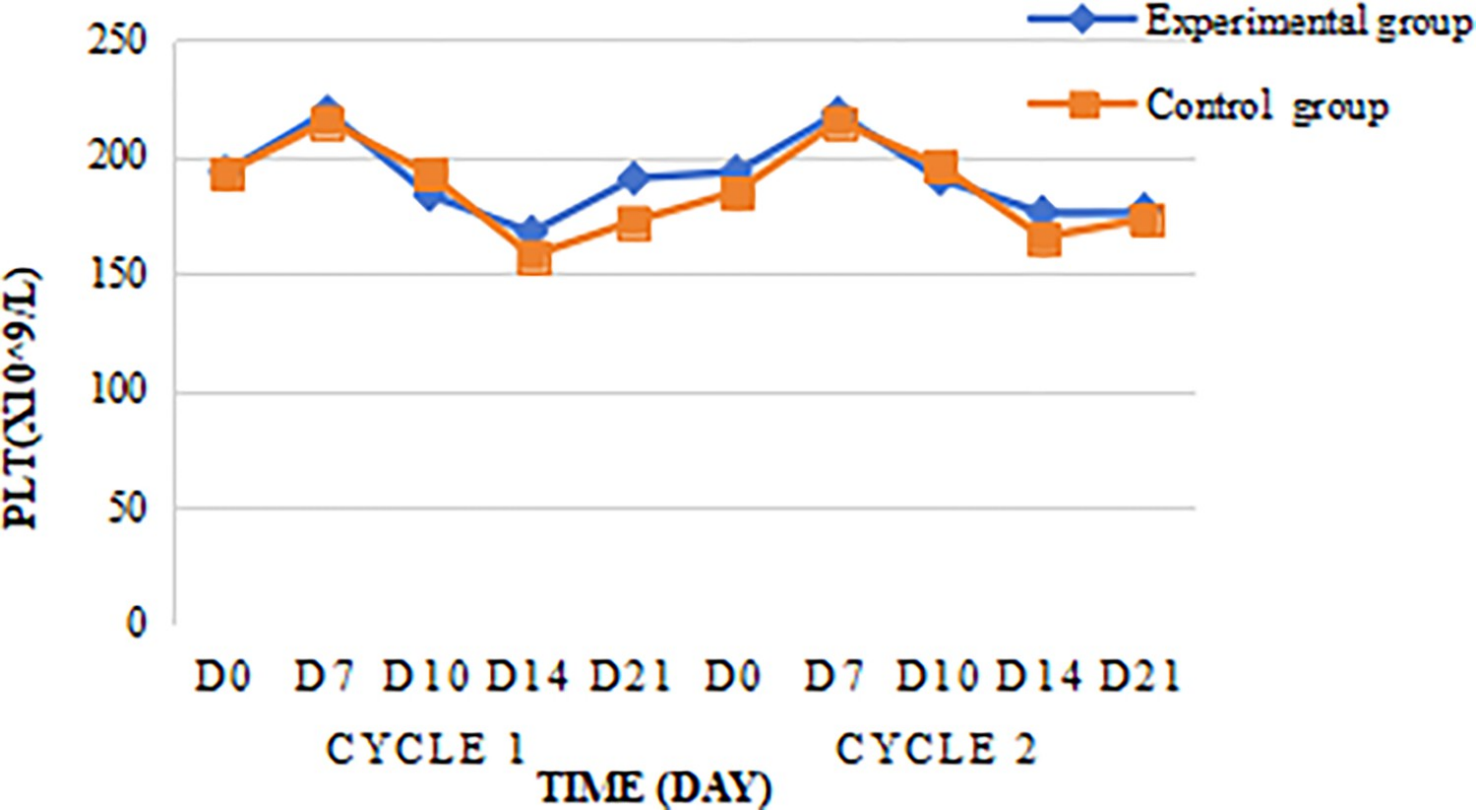
Changes in the platelet count.

The highest platelet value in the experimental group was (218.86 ± 70.77) x 109/L, which occurred on day 7 of the first cycle of chemotherapy, and the highest platelet value in the control group was (214.86 ± 65.63) x 109/L, which also occurred on day 7 of the first cycle of chemotherapy, Platelet values in the experimental and control groups decreased to the lowest value on day 14 and then began to increase. Despite fluctuations in the platelet values within the experimental group, the difference between the two groups was not statistically significant when compared to the control group (P = 0.786) (Fig 4).

**Fig 5:**
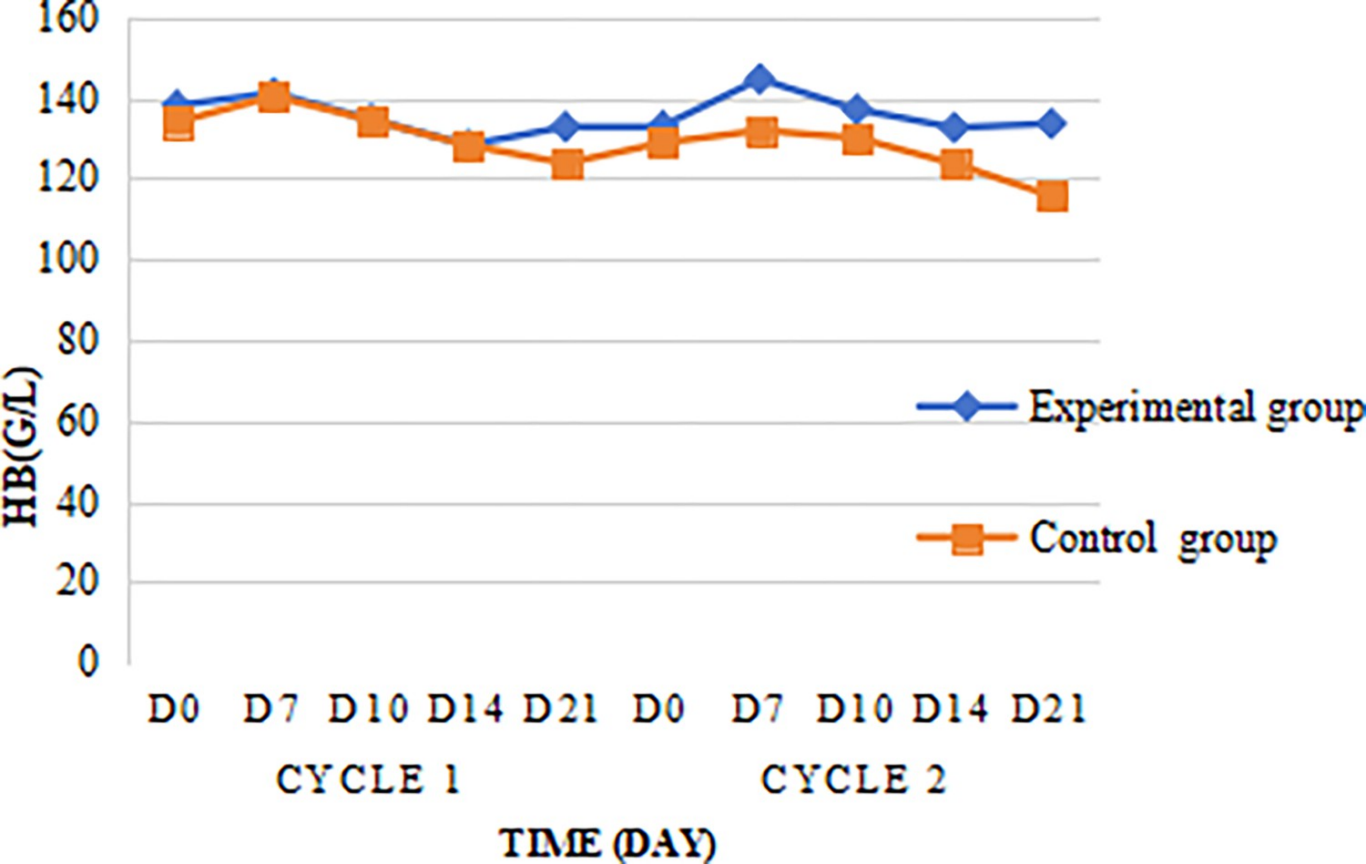
Changes in the hemoglobin.

Both the experimental group and the control group had a trend of first increase and then decrease during the chemotherapy cycle. The difference of hemoglobin (HB) in D7, D10, D14 and D21 was not significant between the two groups (Fig 5), P = 0.257, the difference was not statistically significant.

### The incidence of FN

None of the experimental and control patients developed FN during concurrent chemoradiotherapy.

### Rate of delay in chemotherapy and interruption of radiotherapy

The delay rate of chemotherapy was 2.3% (1/44) in the experimental group and 29.5% (13/44) in the control group, P = 0.001, with a significant difference. One patient in the experimental group had radiotherapy interruption with a rate of 2.3% (1/44), and in the control group, 12 patients had 27.3% (12/44), P = 0.003, a statistically significant difference (Table 4). Analysis of the data showed that prophylactic use of PEG-rhG-CSF significantly improved the timely completion rate of radiotherapy and chemotherapy in patients with locally advanced nasopharyngeal carcinoma.

**Table 4:** Rate of chemotherapy delay and interruption of radiotherapy during concurrent chemoradiotherapy.

	experimental group (n = 44)	control group; matched group (n = 44)	*X* ^*2*^	*P*
The delay rate of chemotherapy	2.3%	29.5%	10.278	0.001
Rates of radiotherapy interruption	2.3%	27.3%	9.026	0.003

### AEs

The incidence of grade I-II oral mucositis in the experimental group was 45.5% (20/44), and the incidence of grade III-IV oral mucositis was 31.8% (14/44). The incidence of grade I -II oral mucositis in the control group was 43.2% (19/44), and the incidence of grade III-IV oral mucositis was 50% (22 /44), P = 0.055. There was no significant difference in the incidence of oral mucositis between the experimental group and the control group (Table 5).

**Table 5:** Incidence of oral mucositis during concurrent chemoradiotherapy.

Grade	Experimental group (n = 44)	Control group (n = 44)	X2	P
			5.795	0.055
I-II	45.5%	43.2%		
III-IV	31.8%	50%		

A total of 15 patients in the experimental group developed bone pain, and the incidence of bone pain in patients was 34.1% (15/44). 13 patients improved on their own without medication, and 2 patients improved on NSAIDs for pain relief. This indicates that the adverse effects of PEG-rhG-CSF are mild and can be tolerated by patients.

**Fig 6:**
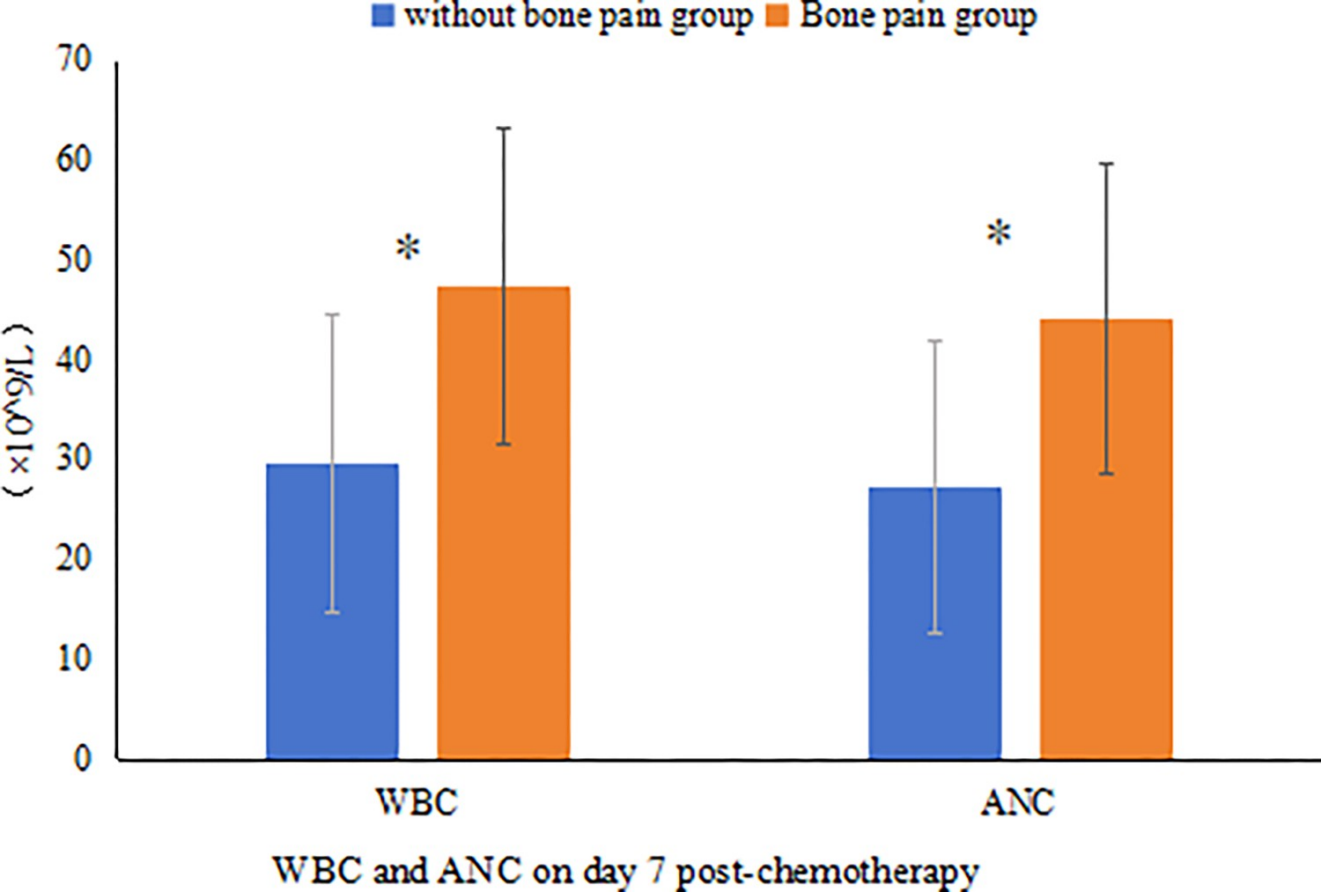
WBC and ANC on day 7 post-chemotherapy.

Further, patients in the experimental group were divided into a bone pain group and a no-bone pain group based on whether they experienced bone pain after subcutaneous injection of PEG-rhG-CSF. The WBC and ANC were compared between the two groups after subcutaneous injection of PEG-rhG-CSF. The study found that the WBC and ANC in both groups were significantly higher than normal values. The patients in the bone pain group had a white blood cell count of (47.45±15.79) x 10^9^/L and a neutrophil count of (44.15±15.49) x 10^9^/L. The patients in the group without bone pain had a white blood cell count of (29.66±14.97) x 10^9^/L and a neutrophil count of (27.22±14.66) x 10^9^/L. And the WBC count and ANC count of the patients in the bone pain group were significantly higher than those of the patients in the group without bone pain, P(WBC) = 0.001, P(ANC) = 0.002 (Fig 6), and the difference was statistically significant. It indicated that the expression level of WBC and ANC after drug administration was correlated with the risk of bone pain, and the higher the value of WBC and ANC, the more likely to develop bone pain.

## Discussion

NPC is one of the common head and neck malignant tumors in southern China. Because of the insidious location of the disease, most nasopharyngeal cancer patients are in locally advanced stages at the time of diagnosis, and concurrent chemoradiotherapy is the standard treatment [33]. Cisplatin is currently the most widely used chemotherapeutic agent in concurrent chemoradiotherapy for nasopharyngeal carcinoma [34]. However, the application of concurrent chemoradiotherapy also brings more adverse effects, among which neutropenia is the main cause of dose reduction and even treatment interruption and delay. rhG-CSF is currently a commonly used drug for the treatment of radiotherapy-induced neutropenia. Because it is mainly cleared through the kidneys and has a short half-life, rhG-CSF needs to be administered for 7–9 consecutive days in order to reach therapeutic dose [22]. PEG-rhG-CSF is the polyethylene glycolized form of rhG-CSF, and the increase in molecular weight makes it possible to achieve the therapeutic dose with only one administration per chemotherapy cycle [35]. The use of prophylactic injections not only reduces the pain of repeated injections and the workload of medical staff, but also prevents the untimely administration of medication in patients with neutropenia.

In this experiment, 88 patients with locally advanced nasopharyngeal cancer were prospectively enrolled. And no patients in the experimental group with prophylactic application of PEG-rhG-CSF developed grade 3/4 neutropenia, and the incidence of grade 3/4 neutropenia in the control group without prophylactic application of PEG-rhG-CSF was 13.6% (6/44), P = 0.026, with the difference being statistically significant; indicating that prophylactic use of PEG-rhG-CSF could reduce the incidence of grade 3/4 neutropenia during synchronized radiotherapy in nasopharyngeal cancer patients, this is similar to the experimental findings of Gu [36]. In Liu F’s study, the incidence of grade 3 neutropenia was 5.8% (3/52) in patients in the PEG group and 26.3% (10/38) in patients in the control group, P = 0.015, a statistically significant difference, and the experimental results proved that accepting prophylactic use of PEG-rhG-CSF reduces the incidence of grade 3/ 4 neutropenia during concurrent chemoradiotherapy for head and neck tumors Grade 3/4 neutropenia, which is consistent with the findings of this experimental study [37]. Nonlinear neutrophil-mediated clearance is the main mode of PEG-rhG-CSF elimination, and after subcutaneous injection of PEG-rhG-CSF, neutrophil counts increase rapidly in an initial phase, reach an apex and enter a phase of rapid nonlinear transition (decrease), and finally enter a phase of slow decrease [38], this is similar to the neutrophil change curve in the present study. This is because the metabolism of PEG-rhG-CSF is "self-regulating", and the concentration of PEG-rhG-CSF in plasma tended to decrease as the neutrophil count in peripheral blood increased [35]. Further analysis of the changes in blood cell counts in the experimental and control groups during the two cycles of chemotherapy revealed that the leukocyte counts and neutrophil counts of the experimental group were higher than those of the control group, and the D7 leukocyte counts and neutrophil counts were much higher than those of the control group. Repeated-measures ANOVA revealed significant differences in ANC between the experimental and control groups (F = 205.04, P < 0.0001). Similarly, there were also highly significant differences among the various time points (F = 109.57, P < 0.0001). Additionally, a significant interaction effect was observed between group and time (F = 87.50, P < 0.0001), indicating that the changes in ANC over time differed significantly between the experimental and control groups, which showed that the prophylactic use of PEG- rhG-CSF can rapidly increase the expression level of leukocytes and neutrophils in vivo, and the effect of elevation is obvious and long-lasting. Tanaka first conducted pharmacokinetic experiments with PEG-rhG-CSF in rats and found that PEG-rhG-CSF prolonged the proliferative activity of neutrophils, thereby reducing the number of administrations [39]. Subsequent van Der Auwera P in vivo studies in healthy subjects confirmed the desirable pharmacokinetic properties and long duration of action of PEG-rhG-CSF [40], this is similar to the findings of the present study. Overstimulation of the neutrophil-producing pathway may cause disruption of the platelet-producing pathway, resulting in lower platelets [41], however, comparing the platelet counts of D7, D10, D14, and D21 in the experimental and control groups in this study, no significant difference was found between the two groups, which is consistent with the findings of Mei Z [42]. To further explore whether PEG-rhG-CSF causes platelet reduction, studies with larger samples are needed.

The on-time, on-demand, and on-schedule administration of radiotherapy has a significant impact on the prognosis of the tumor. In radiation therapy, a conventional fractionation regimen of 1.8–2.0 Gy/dose, once a day, five days a week, is usually chosen because it allows normal tissues to recover from sublethal radiation damage between treatments, but at the same time, the "residual" tumor stem cells may also undergo repopulation or even accelerated repopulation [43]. When the radiotherapy dose accumulates to a certain extent, it can significantly reduce the tumor value increase or even accelerate the tumor value increase [44], If radiotherapy is interrupted or chemotherapy is delayed due to hematological toxicity, it will increase the chance and number of tumor repopulation or even accelerate the repopulation, lowering the local control rate and survival of nasopharyngeal carcinoma, and leading to a poor prognosis of NPC [11, 45]. In this study, the rate of delayed chemotherapy in the experimental group was 2.3%, and the rate of delayed chemotherapy in the control group was 29.5%, P = 0.001, the difference was statistically significant; the rate of interruption of radiotherapy in the experimental group was 2.3%, and the rate of interruption of radiotherapy in the control group was was 27.3%, P = 0.003, the difference was statistically significant. Data analysis showed that prophylactic use of PEG-rhG-CSF could significantly reduce the chemotherapy delay rate and radiotherapy interruption rate during concurrent chemoradiotherapy in patients with locally advanced nasopharyngeal carcinoma, which was similar to the findings of Liu F’s study [37]. Radioactive oral mucositis is a common adverse reaction during radiation therapy for head and neck tumors. Previous studies have shown that more than 90% of patients with nasopharyngeal cancer develop radioactive oral mucositis during concurrent chemoradiotherapy [46]. In this study, it was found that the incidence of grade I-II oral mucositis in the experimental group was 45.5% and grade III-IV oral mucositis was 31.8%, while the incidence of grade I-II oral mucositis in the control group was 43.2% and grade III-IV oral mucositis was 50%, although the incidence of oral mucositis was lower than that of the control group in the experimental group, but the difference was not statistically significant at P = 0.055. PEG-rhG-CSF can reduce systemic or local mucosal inflammatory reactions by stimulating the production and maturation of neutrophils, enhancing the expression and function of neutrophils, thus enhancing neutrophil phagocytosis, chemotaxis, and anti-infective ability [36]. However, there was no significant difference in the incidence of oral mucositis between the experimental group and the control group in this study, which may be attributed to the insufficient number of cases included in this study, and in order to further investigate whether the use of PEG-rhG-CSF can improve the incidence of radiolucent oral mucositis, a clinical study with a larger sample size is needed to analyze it.

Bone pain is the most common adverse reaction to PEG-rhG-CSF, with the back, legs, and buttocks being the most common sites causing pain, and studies have found that pain typically appears within 2 days of administration and lasts for 2–4 days [47], and generally mild symptoms, most patients do not require pain management, and NSAIDs, acetaminophen, corticosteroids, and opioids can better control this type of pain. The specific mechanism by which PEG-rhG-CSF induces bone pain is currently unclear, and possible pathways include bone marrow expansion, sensitization of peripheral injury receptors, modulation of immune function, and direct effects on bone metabolism [48, 49]. The data analysis in this study revealed that the incidence of bone pain in the experimental group was 34.1%, the degree of bone pain was mild, and severe bone pain did not occur. In addition to this, this study found that the neutrophil counts and leukocyte counts of the bone pain group were significantly higher than those of the group without bone pain, and the difference was significant, P(WBC) = 0.001, P(ANC) = 0.002, which indicated that the risk of bone pain was correlated to the expression level of neutrophils and leukocytes, and that the higher the leukocyte and neutrophil values were, the more likely it was to develop bone pain. Moukharskaya’s findings were consistent with the present study. and the higher the value of leukocytes and neutrophils, the more likely to develop bone pain. The findings of Moukharskaya’s study are consistent with the present study [50]. However, only Moukharskaya has reported the relationship between the incidence of bone pain and white blood cell counts, and in order to further investigate the correlation between the incidence of bone pain and blood cell counts, more researchers need to be involved and provide more clinical data.

This study is a single-center, prospective study, due to the limited time of the study, the follow-up time is relatively short, and the difference in tumor local control rate between the experimental group and the control group could not be further analyzed, and it is necessary to increase the sample size and the follow-up time to obtain more complete data. In addition, as the study of Sun Y [5] has reported that there is no significant difference in the risk of myelosuppression during concurrent chemoradiotherapy between the group of induced chemotherapy plus concurrent chemoradiotherapy and the group of concurrent chemoradiotherapy alone, therefore, our study did not further conduct a stratified analysis on whether to conduct induced chemotherapy before concurrent chemoradiotherapy. In this study, the NCCN recommended dose of PEG-rhG-CSF (6mg in a single subcutaneous injection) was used, and it was found that in nasopharyngeal carcinoma, the effect of PEG-rhG-CSF on the elevation of leukocytes and centroblasts was significant, and the clinical efficacy of the PEG-rhG-CSF 3 mg dose group can be further analyzed in the future.

## Conclusions

1. Prophylactic use of PEG-rhG-CSF can reduce the incidence of grade 3 neutropenia during concurrent chemoradiotherapy for locally advanced nasopharyngeal carcinoma.
2. Prophylactic use of PEG-rhG-CSF can rapidly increase the expression levels of white blood cell count and neutrophil count in vivo, and the elevation effect is obvious and long-lasting.
3. Prophylactic application of PEG-rhG-CSF can significantly reduce the rate of delayed chemotherapy and interruption of radiotherapy in patients with nasopharyngeal carcinoma treated with concurrent chemoradiotherapy.
4. The adverse effects of PEG-rhG-CSF were mild, and the risk of bone pain was correlated with the expression levels of leukocytes and neutrophils.

## Supporting information

S1 File The original protocol file. (PDF)

S2 File The translated protocol file. (PDF)

S3 File The CONSORT checklist. (PDF)

S4 File (XLSX)
