# Supplementary material for: Study of PEG-rhG-CSF for the prevention of neutropenia in concurrent chemoradiotherapy for nasopharyngeal carcinoma
Source: PLoS One. 2025 Jan 15;20(1):e0315001. doi: 10.1371/journal.pone.0315001 (PMC11734975; doi:10.1371/journal.pone.0315001)
Supplement: S1 File — (PDF) [file pone.0315001.s001.pdf]

试验方案编号：

版本号：V1.0

版本日期: **2021-04-23**

PEG-rhG-CSF在头颈部鳞癌同步放化疗中一级预防作用的研究

临床研究组长单位：川北医学院附属医院

临床研究主要负责人：杜国波

方案摘要

题目	PEG-rhG-CSF 在头颈部鳞癌同步放化疗中一级预防作用的研究
研究目的	评估聚乙二醇化重组人粒细胞刺激因子（PEG-rhG-CSF）在头颈部鳞癌同期放化疗中预防中性粒细胞减少的有效性和安全性
试验设计	采用前瞻性、单中心、随机对照、干预性研究。入选符合纳入标准的需进行同步放化疗的头颈部鳞癌患者 160 例，随机分为实验组（PEG-rhG-CSF 一级预防）和对照组（不采取一级预防），比较两组同步放化疗期间的血象变化情况、3 / 4 级中性粒细胞减少的发生率、FN 发生率、放化疗中断率和安全性。
入组标准	1) 经病理证实头颈部鳞癌患者<br>2) 年龄：18-70 岁<br>3) 白细胞≥3.0×109/L，中性粒细胞计数≥2.0×109/L，血小板≥100×109/L，血红蛋白≥100g/L<br>4) 卡氏评分≥75<br>5) 无其他恶性肿瘤<br>6) 无血液系统疾病<br>7) 既往无放射治疗史<br>8) 同意入组并自愿签署知情同意书
排除标准	1) 药物过敏患者<br>2) 患者一般身体状况较差，不能忍受治疗；
样本量	共计 160 例患者
主要观察指标	同步放化疗期间 3 / 4 级中性粒细胞减少的发生率
次要观察指标	1) 化疗后第 7、10、14 和 21 天，白细胞、中性粒细胞、血红蛋白和血小板计数的变化；<br>2) 患者 FN 的发生率；<br>3) 放化疗中断率；

	4) 口腔黏膜炎发生率及严重程度；<br>5) 骨痛、乏力的发生率
**安全性评估**	实验室安全检测：包括血红蛋白和血小板计数、肝肾功能。<br>不良事件评估：包括感染和中性粒细胞减少性发热、骨痛等。

目录

PEG-rhG-CSF 在头颈部鳞癌同步放化疗中一级预防作用的研究........1
方案摘要........2
1 研究背景........5
2 研究目的........6
3 受试者选择........6
4 试验设计........7
5 试验药物........7
6 研究程序........7
7 观察指标........9
8 不良事件的记录、报告与处理........10
9 药品管理........11
10 数据记录与管理........11
11 统计分析........12
12 伦理学要求........13
13 方案修改........13
方案签字页........15
附件 1：体力状况 ECOG 评分标准........17

1 研究背景

头颈部恶性肿瘤在全身恶性肿瘤中所占10%左右，且早期头颈部恶性肿瘤对放疗敏感，使得早期头颈部恶性肿瘤预后较好，但头颈部肿瘤大多发现时已晚期，任何单一治疗手段均不理想。在 Maghami E 做的研究中，对于具有手术病理报告且显示阳性切缘或 ENE 的头颈癌患者，在辅助放疗中添加顺铂可使局部区域复发的风险降低约48%，DFS 改善 30%，OS 改善 30%。根据 NCCN 指南：同步放化疗是局部晚期头颈部癌的标准模式。但由于放化疗毒性的累积，常常出现中性粒细胞降低的不良反应，特别是重度中心粒细胞的降低，往往导致同步放化疗无法常规进行，致使放射治疗处方剂量降低，增加了患者肿瘤复发转移的风险。因此如何降低放化疗引起的中性粒细胞减少，是目前头颈部恶性肿瘤患者治疗及改善预后的关键。

聚乙二醇化重组人粒细胞集落刺激因子（PEG-rhG-CSF）是一种长效的重组人粒细胞刺激因子。作用机理是通过诱导中性粒细胞增殖、分化和成熟，增加机体的中性粒细胞水平，从而有助于防止发热性中性粒细胞减少。除此之外，它还提高了成熟中性粒细胞的存活率。与重组人粒细胞集落刺激因子（rhG-CSF）相比，PEG-rhG-CSF 显示出与 rhG-CSF 类似的临床安全性，且 PEG-rhG-CSF 能够更大强度降低血浆清除率，延长半衰期并增加疗效，且免疫原性降低，不良反应小，单剂量 PEG-rhG-CSF 则可以改善中性粒细胞减少症和某些继发症状，从而减轻患者重复注射 rhG-CSF 的痛苦，缩短抗肿瘤治疗时间。有研究表明，对于头颈部肿瘤患者，预防性使用聚乙二醇化重组人粒细胞集落刺激因子（PEG-rhG-CSF）可以降低 ANC 减少及相关性 FN 的发生，有利于按时按预定剂量实施同步放化疗。本研究探讨 PEG-rhG-CSF 对头颈癌同步放化疗时预防中心粒细胞减少的效果及其不良反应，以期为临床用药提供参考。

目前关于 PEG-rhG-CSF 相关的临床研究数据多来自欧美地区，亚洲地区的数据较为有限，且数据较少涉及的头颈部肿瘤。PEG-rhG-CSF 已被多种临床研究证实可预防恶性肿瘤放化疗引起的中心粒细胞降低。如 Liu F 等研究显示预防性使用 PEG-rhG-CSF 可降低住院率和静脉内应用抗生素的比率。在 J Y Zhang 等研究表明在接受多西他赛方案辅助化疗的乳腺癌患者中，应预防性使用 PEG-rhG-CSF 预防中性粒细胞减少和发热性中性粒细胞减少。在 Huang HQ 等的研究分数分析得出：淋巴瘤患者化疗过程中，预防性使用 PEG-rhG-CSF 能够有效降低化疗过程中Ⅲ/Ⅳ度中性粒细胞减少症和 FN 的发生率，确保淋巴瘤患者接受标准剂量化疗，提高治愈率。

2 研究目的

评估聚乙二醇化重组人粒细胞刺激因子 （PEG-rhG-CSF）在头颈部鳞癌同步放化疗中一级预防的作用。

2.1 主要目的

评估聚乙二醇化重组人粒细胞刺激因子（PEG-rhG-CSF）在头颈部鳞癌同期放化疗中预防中性粒细胞减少的有效性和安全性

2.2 次要目的

1) 患者 FN 的发生率（FN：ANC≤0.5×109/L，单次口表体温≥38.3℃或 2 小时内连续两次测量口表体温均≥38.0℃，或 2 小时内连续两次测量腋下温度均≥37.8℃）；
2) 患者 3、4 度 ANC 减少的发生率；
3) 口腔黏膜炎发生率及严重程度；
4) 骨痛、乏力的发生率
5) 放化疗中断率

不良反应评价：

发热，及其他不良事件发生率

3 受试者选择

3.1 入选标准

1) 经病理证实头颈部鳞癌患者
2) 年龄：18-70 岁
3) 白细胞≥3.0×109/L，中性粒细胞计数≥2.0×109/L，血小板≥100×109/L，血红蛋白≥100g/L
4) 卡氏评分≥75
5) 无其他恶性肿瘤
6) 无血液系统疾病
7) 既往无放射治疗史
8) 同意入组并自愿签署知情同意书

3.2 排除标准

1) 药物过敏患者

2) 患者一般身体状况较差，不能忍受治疗；

3.3 退出标准

1）受试者要求退出临床试验；

2）研究者认为患者不适合继续参加本研究；

4 试验设计

采用前瞻性、单中心、干预性研究。实验分为实验和对照组中，两组患者的化疗方案相同，具体如下：顺铂 80mg/m²，放疗同步进行，化疗 21d 为 1 个周期，放疗期间同步两个周期。若为根治性放疗，放疗总剂量规定为 67-72Gy，每周 5 次，每次 1.8-2.2Gy；若为术后辅助放疗，剂量 50-60Gy/25-30 次。实验组在化疗结束 24-48h 内予以 PEG-rhG-CSF 6mg 皮下注射，对照组化疗结束后 24-48h 不预防应用 PEG-rhG-CSF，也不常规预防性使用 G-GSF。如果两组患者在放疗期间白细胞计数＜2.0×109/L 或中性粒细胞计数＜1.0×109/L，则暂停放疗，皮下注射 rhG-CSF，直至白细胞计数≥4.0×109 /L 或中性粒细胞绝对计数≥2.0×109 /L，然后恢复放疗。如果两组患者在第二周期化疗前白细胞计数<3.0×109 /L 或中性粒细胞计数<2.0×109/L，则延迟化疗，皮下注射 rhG-CSF，直至白细胞计数≥3.0×109 /L 或中性粒细胞绝对计数≥2.0×109 /L，恢复化疗。该研究均获患者及其家属知情同意。回访方式以电话、短信、查看病例资料来完成。并通过入选和排除条件来收集有关患者的临床数据. 目标样本量为实体瘤患者 160 例。

5 试验药物

聚乙二醇化重组人粒细胞集落刺激因子（PEG-rhG-CSF，新瑞白）。

6 研究程序

6.1 受试者筛选

患者应签署知情同意书。研究者需要向受试者详细解释知情同意书中涉及的内容，在候选的受试者充分阅读并理解知情同意书的内容后，如其同意参加该临床试验项目，由受试者在知情同意书上签字并注明日期。研究者也需在知情同意书上签名并注明日期。

6.2 入选访视：

- 一般信息：人口学特征、现病史（肿瘤部位、肿瘤分期）、生命体征；
- 体格检查：包括身高、体重、心率、血压、呼吸、脉搏、体表面积、体力分级

以及呼吸、循环等重要系统功能检查；

- 影像学检查：（此项目检查可在化疗前四周内进行）

  正侧位胸片，腹部超声；

  注：如四周内已经进行胸/腹部CT或其他可以代替胸片和超声的影像学检查，可以免做胸片和/或腹部超声。

- 心电图检查：（此项目检查可在化疗前一周内进行）
- 实验室检查：（此项目检查可在化疗前一周内进行）
  - 血常规检查：Hb，WBC，ANC和PLT；
  - 血生化检查：① 肝功能：谷丙转氨酶、谷草转氨酶、碱性磷酸酶、总胆红素、直接胆红素、白蛋白；② 肾功能：血清尿素氮、血清肌酐；
  - 尿常规检查；
- 体力状况评定（ECOG评分）

6.3 研究期间访视

（1）体温监测

- 于试验药物给药当天以及随后每日监测记录最高体温，如体温≥38.0℃，则每隔1小时，连续监测体温2次。

（2）实验室检查

- 血常规：必须为静脉血检测结果，化疗开始后第7/10/14/21天分别查血，并及时记录查血当天的体温及体重变化。
- 血生化：于每周期化疗前进行。必须包括肝、肾功能检查。

（3）不良事件观察

观察并记录受试者的主观感受及皮肤、胃肠道、呼吸、神经、心血管、血液等系统的反应。尤其应详细记录药物可能发生的不良事件，包括乏力、发热、腹泻、关节痛、肌肉疼痛、背痛和感冒样症状的发生、持续时间以及处理措施。

6.4 化疗后院外访视

研究者于患者最后一周期化疗结束出院后20±2日，对患者有无发热、感染等中性粒细胞减少相关症状及肌痛等不良反应进行电话随访。

7 观察指标

7.1 有效性评价：

（1）主要观察终点

同步放化疗期间 3 / 4 级中性粒细胞减少的发生率

（2）次要观察终点

1) 化疗后第 7、10、14 和 21 天，白细胞、中性粒细胞、血红蛋白和血小板计数的变化；
2) 患者 FN 的发生率；
3) 放化疗中断率；
4) 口腔黏膜炎发生率及严重程度；
5) 骨痛、乏力的发生率

7.2 不良事件评价

（1）按 NCI-CTCAE4.0 常见毒性分级标准分为 1、2、3、4、5 级。

（2）与试验药物有关，但 NCI-CTC4.0 标准中没有的不良反应，如感冒样症状（乏力、流涕等）、骨/肌肉/关节痛、注射局部疼痛和注射局部红肿/硬结，应每天观察并记录其出现、消失的时间（化疗周期顺序日、持续时间、程度和处理措施）。

表 1 PEG-rhG-CSF 临床研究流程图

项目	筛选访视	研究期间访视	化疗后院外访视
知情同意书	▲		
受试者一般信息	▲		
体格检查	▲		
影像学检查	▲		
ECOG 评分	▲	▲	
心电图	▲	▲	
血常规	▲	▲	
尿常规	▲	▲	
血生化	▲	▲	
PEG-rhG-CFS 使用方式		▲	
FN/发热		▲	▲

3-4 度中性粒细胞减少		▲	
化疗用药		▲	
化疗延迟/减量		▲	
口腔黏膜炎		▲	▲
乏力、骨痛等不良反应		▲	▲
其他不良事件		▲	▲

8 不良事件的记录、报告与处理

8.1 不良事件的定义

不良事件（Adverse Event），病人或临床试验受试者接受一种药品后出现的不良医学事件，但并不一定与治疗有因果关系。

严重不良事件（Serious Adverse Event），临床试验过程中发生需住院治疗、延长住院时间、伤残、影响工作能力、危及生命或死亡、导致先天畸形等事件。

8.2 不良事件和严重不良事件的记录、报告及处理

受试者在试验过程中发生的任何不良事件必须如实、客观记录在相应的病例报告表（CRF）中，并由医生作相应的判断和必要的处理。

（1）主要记录受试者用药后主观症状及理化检查。准确记录受试者不良事件出现时间、程度、持续时间及处理经过。

（2）明确判断不良事件与药物的关系：不良事件与药物的相关性分为 5 种，见下表：

表 2：不良事件与试验药物的关系

与药物关系	判断标准
肯定有关	● 反应出现的时间符合用药的时间顺序，反应符合试验药物已知的反应类型，停药后改善，重复给药再次出现；
可能有关	● 反应出现的时间符合用药的时间顺序，反应符合试验药物已知的反应类型，病人的临床状态或其它治疗方式也有可能产生该反应；
可能无关	● 反应出现的时间不符合用药的时间顺序，反应不太符合试验药物已知的反应类型，病人临床状态或其它治疗方式也有可能产生该反应；

无　　关	● 反应出现的时间不符合用药的时间顺序，反应有符合非试验药物已知的反应类型，病人的临床状态或其它治疗方式也有可能产生该反应，疾病状态改善或停止其它治疗方式反应消除，重复使用其它治疗方法反应出现；
无法判定	● 反应出现的时间与用药的时间顺序无明确关系，反应与试验药物已知的反应类型相似，同时使用的其它药物也可能引起相同的反应。

注：肯定有关、可能有关、无法判定的不良事件列为不良反应。

（3）报告制度：出现严重不良事件时，研究者填写“严重不良事件报告表”，在 24h 内分别报告国家食品药品监督管理局安全监管司和省级药品监督管理局、申办者及伦理委员会，并在报告上签名及注明日期。

单　位	联系人	电　话
国家市场监督管理总局	安监司	010-88331023
国家卫生健康委员会	医管局	010-68792201
组长单位医学伦理委员会	伦理办公室	0817-2262124

9 药品管理

化疗药物与集落刺激因子都为已上市药物，需要患者按院内正常程序购买使用。

10 数据记录与管理

10.1 数据记录

（1）原始记录：住院受试者的住院病历是临床试验受试者的原始文件，应保存于医院。原始记录应有可溯源性，研究者应严格按临床研究的原则，确保将任何观察与发现正确而完整地记录在病历及研究病历上，保证两者所有数据一致，不得随意更改。作任何更正时不得改变原始记录，只能采取附加叙述并说明理由，由作出更改的研究者签名并注明日期。临床试验中各种实验室数据应记录并将原始报告粘贴在病历及研究病历上。

（2）本项目采用 EDC 系统，数据采集通过新屿信息科技（上海）有限公司的数据系统建立结构化电子病例，进行线上数据管理。

根据项目要求，为保证病人隐私，不应出现病人姓名。患者姓名一律填姓名代码，代码用汉语拼音缩写。

所有选择项目的“□”内用“╳”标注，检验项目因故未查或漏查，应填写：未查；具体用药剂量和时间不明，应填写：不明；填写数字时应将“□”都填满，位数不够的靠右侧填写，左侧空出填“0”。检查项目为阴性项目者填写（—）。不得空项、漏项。研究者应保证所有数据必须与“研究病历”一致。

修改时在原始记录文字的中间划一横线，旁注进行修改，并签署修改者的姓名，注明日期，不得涂抹或覆盖原始记录。

（3）对显著偏离或在临床可接受范围以外的数据(实验室检查项目超过正常值)须加以核实，由参加临床试验的医师做必要的说明。

（4）每一受试者观察疗程结束后，研究者应在 7 个工作日内将“研究病历”的信息以数据形式准确记录在“病例报告表”上，“研究病历”、“病例报告表”和“知情同意书”等试验资料一并交项目负责人审核签字，并存入机构资料档案室，发现问题及时处理并记录。

10.2 数据锁定

确认建立的数据库正确后，由主要研究者、统计分析人员对数据进行锁定。锁定后的数据文件不再做改动。数据锁定之后发现的问题，经确认后在统计分析程序中进行修正。将数据库交统计分析人员按统计计划书要求进行统计分析，并写出统计分析总、分报告，交本试验的主要研究者写出研究报告。

11 统计分析

11.1 统计分析数据集的定义

（1）全分析集（FAS）：对所有经随机化分组，至少使用一次试验药物，且至少有一次疗效随访记录的全部病例进入全分析集（FAS），若有缺失数据采用其缺失资料采用 LOCF 法(last observation carry forward)结转到试验结束。

（2）符合方案数据集（PPS）：对所有经随机化分组，符合试验方案、依从性好、完成全部疗效指标规定填写内容的病例进入符合方案数据集（PPS），并对其疗效进行分析。

（3）安全性数据集（SS）：所有经随机化后至少接受一次试验药物且具有安全性评价数据的病例，构成本研究的安全性数据集（SS）。

（4）基线数据分析采用 FAS 数据集、主要疗效指标对 FAS 和 PPS 数据集同时进行

分析，当两个数据集分析结论一致时，以FAS数据集结论为主。不良事件和不良反应资料均采用SS数据集。

凡具备如下任何一条者，可做剔除处理：

① 误诊者；

② 入选后未用过一次试验用药或研究资料全无记录者

剔除病例应保留备查，除曾已用过药的病例归入SS分析外，其他病例（全未用药者）不进行统计分析。

11.2 统计分析计划书与统计软件

本研究统计由川北医学院附属医院承担，并参与从研究设计、实施至分析总结的全过程。研究方案和病例报告表确定后制定统计分析计划书，采用SPSS26.0统计软件，并在研究过程中根据需要进行必要的修改。数据分析完成后提供统计分析报告。

11.3 统计分析内容

受试者入选数量、脱落和剔病例情况，人口统计学和其它基线特征，化疗中给予PEG-rhG-CSF的剂量、给药时间、给药次数，疗效分析及安全性分析。

11.4 统计分析方法

统计分析计划书由统计人员撰写，在数据库锁定前定稿，以多种表格组成。

统计分析将采用SPSS 26.0统计分析软件进行计算。

12 伦理学要求

临床试验开始前制定试验方案，方案由研究者共同商定并签字，报伦理委员会审批后实施。若本方案在临床试验实际执行过程中出现问题，需要进行修订，修订的试验方案应再次报请伦理委员会批准后实施。如发现涉及试验用药品的重要新资料则必须将知情同意书作书面修改，并送伦理委员会批准后，再次取得受试者同意。

临床试验开始前，研究者必须向受试者提供有关临床试验的详细情况，包括试验性质、试验目的、可能的受益和危险以及受试者的权利和义务等，使受试者充分了解后表示同意，并签署“知情同意书”后方能开始临床试验。

13 方案修改

本方案经伦理委员会批准后，若在实施过程中需要修改，由主要研究者撰写“方案修改说明书”，并签字。重大修改需报请伦理委员会批准后方可实施。

临床试验过程中若因某些客观原因需要病例调整，应经临床试验单位、生物统计

专家同意后进行病例分配的调整。

方案签字页

研究者声明：

我同意严格按照本方案设计和具体规定进行临床试验，仅在获得申办方的批准后对方案中的内容进行修改。

我同意我会亲自执行或监督该临床试验，并且保证在我的单位中协助我执行该临床试验的所有研究人员了解他们在该临床试验中的职责。

在执行该临床试验的过程中，我将严格遵守现行 GCP 和赫尔辛基宣言。并承诺整个试验过程将符合道德上的、伦理上的和科学原理上的要求。

在执行临床试验过程中，我将严格遵守与临床试验相关的所有的法律法规，保护患者的权利和权益。.

我保证我将满足伦理委员会进行审核和批准的要求。

我同意保持足够的和准确的医疗记录，并保证这些医疗记录可随时接受按照相关法律法规进行的稽查和视察。

我同意将及时向伦理委员会报告临床试验活动中的任何改变，以及涉及受试者或其他人员风险的非预期的问题。另外在伦理委员会批准前，我不会在临床试验活动中对临床试验方案作任何修改，除非这些修改是为了在紧急情况下降低患者的风险。

姓　名（正楷）　　　　签　字　　　　日　期

附件 1：体力状况 ECOG 评分标准

ECOG 评分标准	评分
活动能力完全正常，与起病前活动能力无任何差异。	0
能自由走动及从事轻体力活动，包括一般家务或办公室工作，但不能从事较重的体力活动。	1
能自由走动及生活自理，但已丧失工作能力，日间不少于一半时间可以起床活动。	2
生活仅能部分自理，日间一半以上时间卧床或坐轮椅。	3
卧床不起，生活不能自理。	4
死亡。	5
