# Supplementary material for: Study of PEG-rhG-CSF for the prevention of neutropenia in concurrent chemoradiotherapy for nasopharyngeal carcinoma
Source: PLoS One. 2025 Jan 15;20(1):e0315001. doi: 10.1371/journal.pone.0315001 (PMC11734975; doi:10.1371/journal.pone.0315001)
Supplement: S2 File — (PDF) [file pone.0315001.s002.pdf]

Trial Protocol Number：

Version Number: V1.0

Version Date:: 2021-04-23

Study on the role of PEG-rhG-CSF in primary prevention of head and neck squamous carcinoma in concurrent chemoradiotherapy

Sponsoring Institution：Affiliated Hospital of North Sichuan Medical College

Principal Investigator：Guobo Du

Abstract

**Title**	Study on the role of PEG-rhG-CSF in primary prevention of head and neck squamous carcinoma in concurrent chemoradiotherapy
**Purpose of the Study**	To evaluate the efficacy and safety of polyethylene glycol-conjugated recombinant human granulocyte colony-stimulating factor (PEG-rhG-CSF) in preventing neutropenia during concurrent chemoradiotherapy for squamous cell carcinoma of the head and neck.
**Study Design**	A prospective, single-center, randomized controlled, interventional study. A total of 160 patients with squamous cell carcinoma of the head and neck who meet the inclusion criteria and are undergoing concurrent chemoradiotherapy will be randomly divided into the experimental group (PEG-rhG-CSF primary prevention) and the control group (no primary prevention). The study will compare the hematological changes, incidence of grade 3/4 neutropenia, febrile neutropenia (FN), treatment interruption rate, and safety between the two groups during concurrent chemoradiotherapy.

**Inclusion Criteria**	1) Patients with pathologically confirmed squamous cell carcinoma of the head and neck.<br>2) Age: 18-70 years.<br>3) White blood cell count $\geq 3.0\times10^9$/L, neutrophil count $\geq 2.0\times10^9$/L, platelet count $\geq 100\times10^9$/L, hemoglobin ≥100g/L.<br>4) Karnofsky Performance Status (KPS) ≥75.<br>5) No other malignant tumors.<br>6) No hematological disorders.<br>7) No history of radiotherapy.<br>8) Willing to participate and sign the informed consent form.
**Exclusion Criteria**	1) Patients with drug allergies.<br>2) Patients with poor general physical condition who cannot tolerate treatment.
**Sample Size**	A total of 160 patients.
**Primary**	The incidence of grade 3/4 neutropenia during concurrent chemoradiotherapy.

**Outcome Measure**	
**Secondary Outcome Measures**	1) Changes in white blood cell, neutrophil, hemoglobin, and platelet counts on days 7, 10, 14, and 21 after chemotherapy；<br>2) incidence of FN；<br>3) treatment interruption rate；<br>4) incidence and severity of oral mucositis；<br>5) incidence of bone pain and fatigue
**Safety Assessment**	Laboratory safety tests: including hemoglobin and platelet counts, liver and kidney function.<br>Adverse event assessment: including infections and neutropenic fever, bone pain, etc.

Catalogs

Abstract 2
1 Background 6
2 Purpose of the Study 7
3 Selection of Subjects 7
4 Study Design 9
5 Trial Medication 9
Polyethylene Glycol-Recombinant Human Granulocyte Colony-Stimulating Factor (PEG-rhG-CSF, brand name: Xin Ruibai). 9
6 Study Procedures 9
7 Observational Indicators 12
8 Recording, Reporting, and Management of Adverse Events: 14
9 Drug Management: 16
10 Data Recording and Management: 16
11 Statistical Analysis 18
12 Ethical Requirements 20
13 Protocol Amendments 20
Attachment 1: ECOG Performance Status Score Standard 22

1 Background

Malignant tumors of the head and neck account for approximately 10% of all malignancies in the body. Early-stage head and neck malignancies are sensitive to radiotherapy, leading to better prognosis. However, most head and neck tumors are diagnosed at an advanced stage, and no single treatment modality is ideal. In a study by Maghami E, the addition of cisplatin to adjuvant radiotherapy for head and neck cancer patients with surgical pathology reports showing positive surgical margins or extranodal extension (ENE) reduced the risk of local-regional recurrence by approximately 48%, improved disease-free survival (DFS) by 30%, and overall survival (OS) by 30%. According to the NCCN guidelines, concurrent chemoradiotherapy is the standard modality for locally advanced head and neck cancer. However, due to the cumulative toxicity of chemoradiotherapy, adverse reactions such as neutropenia, especially severe neutropenia, often occur, which often prevents the routine administration of concurrent chemoradiotherapy, resulting in reduced prescribed doses of radiotherapy and increased risk of tumor recurrence and metastasis. Therefore, reducing neutropenia induced by chemoradiotherapy is crucial for the treatment and prognosis improvement of patients with head and neck malignancies.

Pegylated recombinant human granulocyte colony-stimulating factor (PEG-rhG-CSF) is a long-acting recombinant human granulocyte stimulating factor. Its mechanism of action involves inducing proliferation, differentiation, and maturation of neutrophils to increase the body's neutrophil levels, thereby helping to prevent febrile neutropenia. In addition, it enhances the survival rate of mature neutrophils. Compared with recombinant human granulocyte colony-stimulating factor (rhG-CSF), PEG-rhG-CSF demonstrates similar clinical safety and can reduce plasma clearance, prolong half-life, and increase efficacy with lower immunogenicity and fewer adverse reactions. A single dose of PEG-rhG-CSF can improve neutropenia and certain secondary symptoms, thereby reducing the burden of repeated rhG-CSF injections for patients and shortening the duration of antitumor treatment. Studies have shown that prophylactic use of PEG-rhG-CSF in patients with head and neck tumors can reduce the occurrence of absolute neutrophil count (ANC) reduction and related febrile neutropenia (FN), facilitating the timely and scheduled administration of concurrent chemoradiotherapy. This study explores the efficacy and adverse reactions of PEG-rhG-CSF in preventing neutropenia during concurrent chemoradiotherapy for head and neck cancer, aiming to provide a reference for clinical medication.

Currently, clinical research data on PEG-rhG-CSF are mostly from Europe and North America, with limited data from Asia, and few studies involve head and neck tumors. PEG-rhG-CSF has been proven by multiple clinical studies to prevent neutropenia induced by chemoradiotherapy for malignancies. For example, Liu F et al. showed that prophylactic use of PEG-rhG-CSF could reduce hospitalization rates and the ratio of intravenous antibiotic application. J Y

Zhang et al. indicated that prophylactic use of PEG-rhG-CSF should be used in breast cancer patients receiving docetaxel-based adjuvant chemotherapy to prevent neutropenia and febrile neutropenia. A fractional analysis of Huang HQ et al.'s study concluded that prophylactic use of PEG-rhG-CSF in lymphoma patients during chemotherapy can effectively reduce the incidence of grade III/IV neutropenia and FN, ensuring that lymphoma patients receive standard-dose chemotherapy and improving cure rates.

2 Purpose of the Study

To evaluate the primary prophylactic role of Polyethylene Glycol-Recombinant Human Granulocyte Colony-Stimulating Factor (PEG-rhG-CSF) in concurrent chemoradiotherapy for squamous cell carcinoma of the head and neck.

2.1Primary Objective

To assess the efficacy and safety of Polyethylene Glycol-Recombinant Human Granulocyte Colony-Stimulating Factor (PEG-rhG-CSF) in preventing neutropenia during concurrent chemoradiotherapy for squamous cell carcinoma of the head and neck.

2.2 Secondary Objectives

1) Incidence of FN (FN: ANC≤0.5×10^9/L, single oral temperature ≥38.3℃ or two measurements within 2 hours ≥38.0℃, or two measurements within 2 hours ≥37.8℃),
2) incidence of grade 3 and 4 ANC reduction；
3) incidence and severity of oral mucositis；
4) incidence of bone pain and fatigue,
5) treatment interruption rate
6) adverse reaction evaluation：fever, and other adverse event incidence

3 Selection of Subjects

3.1 Inclusion Criteria

1) Patients with pathologically confirmed squamous cell carcinoma of the head and neck.
2) Age: 18-70 years
3) WBC≥3.0×109/L，ANC≥2.0×109/L，PLT≥100×109/L，HB≥100g/L
4) Karnofsky Performance Status (KPS) ≥75.
5) No other malignant tumors.
6) No hematological disorders.
7) No history of radiotherapy.
8) Willing to participate and sign the informed consent form.

3.2 Exclusion Criteria

1) Patients with drug allergies.
2) Patients with poor general physical condition who cannot tolerate treatment.

3.3 Withdrawal Criteria

1) Subjects requesting to withdraw from the clinical trial;

2）Investigators deeming patients unsuitable to continue participation in this study.

4 Study Design

A prospective, single-center, interventional study. The experiment is divided into an experimental and a control group, with both groups receiving the same chemotherapy regimen, as follows: Cisplatin 80mg/m², with concurrent radiotherapy, the chemotherapy cycle is 21 days, two cycles concurrent with radiotherapy. If radical radiotherapy is indicated, the total radiotherapy dose is prescribed as 67-72Gy, five times per week, at 1.8-2.2Gy per fraction; if postoperative adjuvant radiotherapy is indicated, the dose is 50-60Gy/25-30 fractions. The experimental group will receive PEG-rhG-CSF 6mg subcutaneous injection within 24-48 hours after chemotherapy, while the control group will not receive PEG-rhG-CSF prophylactically and will not routinely use G-CSF. If patients in either group have a white blood cell count $<2.0\times10^9$/L or neutrophil count $<1.0\times10^9$/L during radiotherapy, radiotherapy will be paused, and subcutaneous rhG-CSF will be administered until the white blood cell count is $\geq4.0\times10^9$/L or the absolute neutrophil count is $\geq2.0\times10^9$/L, after which radiotherapy will resume. If patients in either group have a white blood cell count $<3.0\times10^9$/L or neutrophil count $<2.0\times10^9$/L before the second cycle of chemotherapy, chemotherapy will be delayed, and subcutaneous rhG-CSF will be administered until the white blood cell count is $\geq3.0\times10^9$/L or the absolute neutrophil count is $\geq2.0\times10^9$/L, after which chemotherapy will resume. This study has obtained informed consent from patients and their families. Follow-up will be completed through telephone calls, text messages, and medical record reviews, and clinical data on patients will be collected based on the inclusion and exclusion criteria. The target sample size is 160 patients with solid tumors.

5 Trial Medication

Polyethylene Glycol-Recombinant Human Granulocyte Colony-Stimulating Factor (PEG-rhG-CSF, brand name: Xin Ruibai).

6 Study Procedures

6.1 Subject Screening

Patients should sign the informed consent form. Investigators need to explain the content of the informed consent form in detail to the subjects. After the candidates have fully read and understood the content of the informed consent form and agree to participate in the clinical trial project, they will sign and date the informed consent form. Investigators should also sign and date the informed consent form.

6.2Inclusion Visit：

● General information: Demographic characteristics, present medical history (tumor location, tumor staging), vital signs;

● Physical examination: Including height, weight, heart rate, blood pressure, respiration, pulse, body surface area, performance status, and functional examination of important systems such as respiratory and circulatory systems;

● Imaging examinations: (This project can be performed within four weeks before chemotherapy)

Posteroanterior chest X-ray, abdominal ultrasound;

Note: If chest/abdominal CT or other imaging examinations that can replace chest X-ray and ultrasound have been performed within four weeks, chest X-ray and/or abdominal ultrasound may be waived

.● Electrocardiogram (ECG) examination: (This project can be performed within one week before chemotherapy)

● Laboratory tests: (This project can be performed within one week before chemotherapy)

◆ Complete blood count: Hb, WBC, ANC, and PLT;

◆ Biochemical tests: 1) Liver function: Alanine aminotransferase (ALT), Aspartate aminotransferase (AST), Alkaline phosphatase (ALP), Total bilirubin (TBIL), Direct bilirubin (DBIL), Albumin (ALB); 2) Renal function: Blood urea nitrogen (BUN), Serum creatinine (Scr);

◆ Urinalysis;；

- Performance status assessment (ECOG scoring).

6.3 Study Visits

（1）Temperature monitoring

- Record the highest temperature on the day of trial medication administration and subsequently monitor and record the highest temperature daily. If the temperature is ≥38.0℃, monitor the temperature every hour for two consecutive measurements.

（2）Laboratory tests

- Complete blood count: Must be venous blood test results, blood tests should be conducted on days 7/10/14/21 after the start of chemotherapy and record the temperature and weight changes on the day of blood testing.
- Biochemical tests: Conducted before each cycle of chemotherapy. Must include liver and kidney function tests.

（3）Adverse event observation

Observe and record the subjective feelings of the subjects and reactions in the skin, gastrointestinal, respiratory, nervous, cardiovascular, hematopoietic, and other systems. Particularly, detailed records should be kept of adverse events that may occur with the medication, including fatigue, fever, diarrhea, arthralgia, muscle pain, back pain, and flu-like symptoms, occurrence, duration, and management measures.

6.4 Post-Chemotherapy Outpatient Visits:

Investigators will conduct telephone follow-ups with patients 20±2 days after discharge from the last cycle of chemotherapy to assess for symptoms related to neutropenia such as fever and infections, as well as adverse reactions such as myalgia.

7 Observational Indicators

7.1 Efficacy Evaluation

（1） Primary endpoint

The incidence of grade 3/4 neutropenia during concurrent chemoradiotherapy.

（2） Secondary endpoints

1)Changes in white blood cell, neutrophil, hemoglobin, and platelet counts on days 7, 10, 14, and 21 after chemotherapy;

2)Incidence of FN;

3) Treatment interruption rate;

4) Incidence and severity of oral mucositis;

5) Incidence of bone pain and fatigue.

7.2Adverse Event Evaluation:

（1） Adverse events are graded according to the NCI-CTCAE 4.0 common toxicity grading standards into grades 1, 2, 3, 4, and 5.

（2） Adverse reactions related to the trial medication but not included in the NCI-CTC 4.0 standards, such as flu-like symptoms (fatigue, rhinorrhea, etc.), bone/muscle/joint pain, injection site pain, and injection site redness/induration, should be observed and recorded daily for their appearance, disappearance time (chemotherapy cycle sequence day, duration, severity, and management measures).

Table:1 Clinical Study Flowchart of PEG-rhG-CSF

Item	Screening	Study Visit	Post-Chemotherap

	**Visit**		**y Outpatient Visit**
Informed Consent	▲		
Subject General Information	▲		
Physical Examination	▲		
Imaging Examinations	▲		
ECOG Score	▲	▲	
Electrocardiogram	▲	▲	
Blood Routine	▲	▲	
Urine Routine	▲	▲	
Blood Biochemistry	▲	▲	
PEG-rhG-CSF Administration		▲	
FN/Fever		▲	▲
Grade 3-4 Neutropenia		▲	
Chemotherapy Medication		▲	
Chemotherapy Delay/Dosage Reduction		▲	
Oral Mucositis		▲	▲
Fatigue, Bone Pain, and Other Adverse Reactions		▲	▲
Other Adverse Events		▲	▲

8 Recording, Reporting, and Management of Adverse Events:

8.1 Definition of Adverse Events:

Adverse Event (AE), any untoward medical occurrence in a patient or clinical trial subject administered a pharmaceutical product, which does not necessarily have a causal relationship with the treatment.

Serious Adverse Event (SAE), any untoward medical occurrence that, at any dose, results in any of the following outcomes during the clinical trial: requires inpatient hospitalization or prolongation of existing hospitalization, causes disability, is life-threatening, or results in death, or requires intervention to prevent permanent impairment or damage to a body structure or a body function, or congenital anomaly/birth defect.

8.2 Recording, Reporting, and Management of Adverse and Serious Adverse Events:

Any adverse events occurring during the trial must be truthfully and objectively recorded in the corresponding case report form (CRF) and assessed and managed by a physician as necessary.

（1） Mainly record the subjective symptoms of the subjects after medication and physical examinations. Accurately record the time of occurrence, severity, duration, and management process of adverse events.

（2）Clearly judge the relationship between adverse events and the medication: The relationship between adverse events and medication is divided into five types, as shown in the table below:

Table 2: Relationship between Adverse Events and Trial Medication

**Relationship with Medication**	**Criteria for Assessment**

Definitely Related	The appearance time of the reaction is consistent with the medication administration sequence, and the reaction is consistent with the known reaction types of the trial medication, with improvement after discontinuation of the medication and reoccurrence upon readministration;
Possibly Related	The appearance time of the reaction is consistent with the medication administration sequence, and the reaction is consistent with the known reaction types of the trial medication, but the clinical condition of the patient or other treatments could also cause the reaction;
Possibly Unrelated	The appearance time of the reaction is inconsistent with the medication administration sequence, and the reaction is not consistent with the known reaction types of the trial medication, but the clinical condition of the patient or other treatments could also cause the reaction;
Unrelated	The appearance time of the reaction is inconsistent with the medication administration sequence, and the reaction is consistent with non-trial medication known reaction types, and the disease state improves or other treatments are stopped, and the reaction disappears, and the reaction occurs with the readministration of other treatment methods;
Unable to Determine	The appearance time of the reaction has no clear relationship with the medication administration sequence, the reaction is similar to the known reaction types of the trial medication, and other medications used at the same time may also cause the same reaction.

Note: Adverse events that are definitely related, possibly related, or unable to determine are considered adverse reactions.

（3）Reporting System: In the event of a serious adverse event, the investigator shall fill out the "Serious Adverse Event Report Form," and

within 24 hours, report to the National Food and Drug Administration's Safety Supervision Department, the provincial drug administration, the sponsor, and the ethics committee, respectively. The investigator must sign and date the report.

Unit	Contact	Phone Number
State Administration for Market Regulation	Safety Supervision Department	010-88331023
National Health Commission	Medical Administration	010-68792201
Leading Institution's Medical Ethics Committee	Ethics Office	0817-2262124

9 Drug Management:

Chemotherapy medications and colony-stimulating factors are all marketed drugs that require patients to purchase and use through the hospital's normal procedures.

10 Data Recording and Management:

10.1 Data Recording:

(1) Original records: The medical records of inpatients participating in the clinical trial are the original documents and should be preserved in the hospital. Original records should be traceable. Investigators should strictly follow clinical trial principles to ensure that all observations and findings are correctly and completely recorded in the medical records and research medical records, ensuring consistency between the two and not altering the data arbitrarily. Any corrections should not change the original records but should be made by adding a narrative and explaining the reason, signed by the investigator who made the change and dated. All laboratory data from the clinical trial should be recorded and the original reports pasted in the medical records and research medical

records.

（2）This project uses an EDC system for data collection through the data system established by Xinyu Information Technology (Shanghai) Co., Ltd., for online data management.

According to project requirements, in order to protect patient privacy, patient names should not appear. Instead, patient names should be filled in with name codes, which are abbreviations in pinyin (Chinese phonetic alphabet).

All selection items in the "□" should be marked with "╳", if the test item is not tested or missed, it should be filled in as: not tested; if the specific dosage and time of medication are unclear, it should be filled in as: unclear; when filling in numbers, the "□" should be filled completely, with the insufficient digits filled on the right and the left side filled with "0". Negative test items should be filled in as (－). No items should be left blank or missed. Investigators should ensure that all data must be consistent with the "research medical records".

When modifying, a line should be drawn through the middle of the original record text, with the modification made alongside and signed by the modifier, with the date noted. Original records should not be smeared or covered.

（3） Significant deviations or data outside the clinical acceptable range (laboratory test items exceeding normal values) must be verified, with explanations provided by the physician participating in the clinical trial.

（4） After the completion of the observation course for each subject, the investigator should accurately record the information from the "research medical records" in the form of data on the "case report form" within 7 working days, and the "research medical records", "case report form", and "informed consent form" and other trial materials should be reviewed and signed by the project leader and stored in the institutional archive room, with any problems promptly dealt with and recorded.

10.2 Data Locking

After confirming that the established database is correct, the principal investigator and the statistical analyst will lock the data. The locked data file will not be changed. Any problems found after data locking will be corrected in the statistical analysis program. The database will be handed over to the statistical

analyst to perform statistical analysis according to the statistical plan and to write the statistical analysis report, which will be submitted to the principal investigator of this trial to write the trial report.

11 Statistical Analysis

11.1 Definition of Statistical Analysis Data Sets:

(1) Full Analysis Set (FAS): All cases that have been randomized, have used the trial medication at least once, and have at least one efficacy follow-up record will be included in the Full Analysis Set (FAS). If there are missing data, the last observation carry-forward (LOCF) method will be used to impute the data until the end of the trial.

(2) Per Protocol Set (PPS): All cases that have been randomized, comply with the trial protocol, have good compliance, and complete all efficacy indicator specifications will be included in the Per Protocol Set (PPS), and their efficacy will be analyzed.

(3) Safety Set (SS): All cases that have been randomized and have received the trial medication at least once and have safety evaluation data constitute the Safety Set (SS) of this study.

(4) Baseline data analysis will use the FAS data set, and the main efficacy indicators will be analyzed for both FAS and PPS data sets. When the conclusions of the two data sets are consistent, the conclusions of the FAS data set will be the primary reference. Safety and adverse event data will be analyzed using the SS data set.

Any of the following cases may be excluded:

① Misdiagnosed cases;；

② Cases that have not used the trial medication even once after selection or have no study records.

Excluded cases should be kept for reference, and in addition to cases that have used medication being included in the SS analysis, other cases (those who

have not used any medication) will not be statistically analyzed.

11.2 Statistical Analysis Plan and Statistical Software:

The statistics of this study will be undertaken by The Affiliated Hospital of North Sichuan Medical College, which will participate in the entire process from study design, implementation to analysis and summary. After the study plan and case report form are determined, a statistical analysis plan will be developed, using SPSS 26.0 statistical software, and necessary modifications will be made as needed during the study. A statistical analysis report will be provided after the data analysis is completed.

11.3 Content of Statistical Analysis:

The number of subjects included, dropouts, and excluded cases, demographic statistics, and other baseline characteristics, dosage, administration time, and frequency of PEG-rhG-CSF given during chemotherapy, efficacy analysis, and safety analysis.

11.4 Statistical Analysis Methods:

The statistical analysis plan will be written by the statistical personnel and finalized before the database is locked, consisting of various tables.

Statistical analysis will be performed using SPSS 26.0 statistical analysis software.

12 Ethical Requirements

Before initiating the clinical trial, a trial protocol is developed and submitted to the ethics committee for approval before implementation. If issues arise during the actual execution of the clinical trial that necessitate revisions to the protocol, the revised protocol shall once again be submitted to the ethics committee for approval before implementation. In the event that significant new information concerning the investigational medicinal product is discovered, the informed consent form must be amended in writing, submitted to the ethics committee for approval, and then re-obtained from the subjects with their consent.

Before the start of the clinical trial, researchers must provide detailed information about the clinical trial to the subjects, including the nature of the trial, the purpose, potential benefits and risks, and the rights and obligations of the subjects, ensuring that the subjects fully understand and consent before starting the clinical trial by signing the "Informed Consent Form".

13 Protocol Amendments

After this protocol is approved by the ethics committee, if modifications are needed during the implementation process, the principal investigator will write an "Amendment Description" and sign it. Major modifications need to be approved by the ethics committee before implementation.

During the clinical trial process, if adjustments to the case are needed due to certain objective reasons, they should be agreed upon by the clinical trial unit and biostatistics experts before adjusting the case allocation.

Signature Page of the Protocol:

Statement by the Investigator:

I agree to conduct the clinical trial strictly in accordance with the design and specific provisions of this protocol, and to modify the contents of the protocol only after obtaining approval from the sponsor.

I agree to personally execute or supervise this clinical trial and ensure that all researchers assisting me in executing this clinical trial at my institution understand their responsibilities within the trial.

During the execution of this clinical trial, I will strictly adhere to the current Helsinki Declaration and commit to conducting the entire trial in accordance with moral, ethical, and scientific principles.

In conducting the clinical trial, I will strictly abide by all laws and regulations related to clinical trials and protect the rights and interests of patients.
I guarantee that I will meet the requirements for review and approval by the ethics committee.

I agree to maintain sufficient and accurate medical records and ensure that these records are available for audits and inspections conducted in accordance with relevant laws and regulations at any time.

I agree to promptly report any changes in clinical trial activities and any unexpected issues involving risks to subjects or other personnel to the ethics committee. Additionally, I will not make any modifications to the clinical trial protocol in clinical trial activities prior to approval by the ethics committee, unless these modifications are necessary to reduce patient risk in emergency situations.

______________________	______________________	______________________
Name (Kai letters)	Signature	Date

Attachment 1: ECOG Performance Status Score Standard

Activity Level	Score
Fully active, able to carry on all normal activity without restriction	0
Restricted in physically strenuous activity but ambulatory and able to carry out work of a light or sedentary nature, e.g., light house work, office work	1
Ambulatory and capable of all self-care but unable to carry out any work activities. Up 50% of waking hours	2
Capable of only limited self-care, confined to bed or chair 50% or more of waking hours	3
Completely disabled. Cannot carry on any self-care. Bedbound or chairbound	4
Dead	5
